# Practical Remarks on the Use of Carbonate of Lead, in the Form of White Paint, in the Treatment of Burns and Scalds

**Published:** 1845-08

**Authors:** Samuel D. Gross

**Affiliations:** Professor of Surgery in the Medical Institute of Louisville Ky.


*Practical Remarks on the Use of Carbonate of Lead, in the form of White Paint, in the Treatment of Burns and Scalds.* By S. D. Gross, M. D., Professor of Surgery in the Medical Institute of Louisville, Ky.

Burns and scalds are of daily occurrence. To manage them properly must, therefore, be an object of primary importance; and yet there are no injuries which are approached by the practitioner with such feelings of doubt and apprehension. The reason of this is obvious. The whole subject has been invested from time immemorial with a sort of mystery; every writer has his peculiar classification; and the treatment even at the present day, is confessedly of the most opposite and diversified character. In fact, there is scarcely an article of the materia medica that has not had its advocates in some stage or other of these lesions.

It is not my intention, in this paper, to speak of the various forms of burns, the phenomena by which they are characterized, or the effects to which they may give rise, when severe, neglected, or injudiciously managed. This has been so often done that anything that might be said would, of necessity, be a mere repetition of what may be found in our surgical works. My object is to call attention to a mode of treatment, of the value of which few physicians seem to be aware. I allude to the use of carbonate of lead, in the form of white paint. It is not unknown to me that this article has occasionally been resorted to by the common people as an application to burns and scalds; but no writer, so far as my information extends, has treated of it *ex professo.* My attention was first directed to the subject about two years ago, by Dr. Somerby, an eminent dentist of this city, who, in manufacturing porcelain teeth, and in consequence of often burning his hands and fingers, always finds prompt relief from the employment of white lead. Since that time I have constantly used this substance in my practice, and now look on it as the most valuable therapeutic agent of the kind we possess.

One of the most important indications to be fulfilled in the treatment of this class of injuries is, to prevent the contact of the atmosphere. If this can be accomplished, the pain soon subsides, and unless the burn be very extensive, or the patient unusually irritable, or debilitated by previous disease, recovery is almost certain. This object has been attempted to be attained by various means, of which the most efficient are carded cotton and the liniment of lime-water, or Carron-oil, as it is sometimes called from its having been so much employed at the iron-works of that name in Scotland. Lately Mr. Rhind, of England, has recommended for the same purpose a solution of gum arabic, repeated coats of which are applied, so as to form a complete covering to the affected surface. In several cases in which he tried this method, prompt relief seems to have followed.

It is difficult to determine whether the white lead produces its beneficial effects simply by preventing the injurious impression of the atmosphere, or whether it also at the same time obtunds the nervous sensibility. The probability is that it acts in both ways. However this may be, I know of no application which so speedily allays pain, prevents the formation of vesicles, and puts a stop to inflammation. I have seen patients, literally agonized with suffering, become perfectly composed, and even cheerful, within a few minutes after the burned surface was thoroughly painted. In fact, it generally acts like a charm.

The best mode of applying the white lead is by means of a large camel's hair pencil, or, what answers equally well, and can always be readily obtained, a soft muslin or linen mop. With this the affected surface is covered with a layer sufficiently thick to conceal it from view. If vesicles exist, their contents must be evacuated with a fine needle, and the part dried with a soft sponge, or carded cotton, otherwise the lead will not adhere. The dressing is completed by covering the painted surface with a piece of old muslin or linen, and confining it with a moderately light roller. I have on several occasions used carded cotton instead of muslin, but have usually found it objectionable, on account of its tendency to imbibe the secretions and keep up too much heat. For the same reason, when the burn is deep, and the discharge likely to be considerable, the thin compress here recommended should be perforated with numerous holes, so as to afford a proper drain. The number of dressings, or the frequency with which they are repeated, must depend upon circumstances. In superficial burns, one application will often be sufficient; if the lesion, however, involve the deeper layers of the skin, or extend entirely through its substance, it should be renewed at least once in the twenty-four hours. Whenever the dressings become saturated with secretions, the part is rendered painful, and the system irritable. In mild cases, the paint and epidermis form a sort of dry incrustation, which usually drops off in three or four days, leaving the surface beneath entirely well, or of a slightly excoriated appearance.

The lead of the shops is very stiff, and consequently unfit for use. Before it is laid on, it should be well mixed with linseed oil, in the proportion of about one ounce of the former to a drachm of the latter, or until it is of the consistence of thick cream. When the linseed oil is not at hand, olive oil may be advantageously used as a substitute.

It is not merely to the milder forms of burns and scalds that this remedy is applicable; it may be beneficially employed whatever be the extent or depth of the injury. When the skin is converted into a cinder or an eschar, it matters not what we use; the contact of the atmosphere can do no harm, and the great indication, as far as the local treatment is concerned, is to promote the detachment of the slough, and establish granulation. After this process has fairly commenced, or even before, the paint again constitutes, according to my experience, the most soothing and eligible application that I know. In the case of a negro wowan, 16 years of age, who had a sloughing burn, extending, on the one hand, from the umbilicus to the lower part of the face, and, on the other, as far out on each side as on a line with the axilla, the only application I used was the white lead paint, applied, at first, every other day, and afterwards, as the discharges became more abundant, once a day, until the cicatrization was completed. The period occupied by this process was upwards of five weeks; and, although the quantity of diluted lead consumed was upwards of a quart, no unpleasant effects whatever ensued from its application. It is particularly important in all cases of this kind that the dressings be frequently renewed: that is, at least once in the twenty-four hours, otherwise they will become stiff, offensive, and a source of more or less suffering. When the sore is very large, it is best to expose only a part of it at a time.

The external use of white lead is regarded by many practitioners as extremely dangerous, on account of the risk of absorption; I have never, however, in a solitary instance witnessed any bad effects from it. In some of the cases in which I have either employed it myself or seen it used by others, the injury occupied a large extent of surface, and penetrated nearly,—in some places entirely,—the whole thickness of the skin. I am convinced, therefore, that the danger is greatly overrated, if not altogether imaginary. Granting, however, that the application is not devoid of danger, all unpleasant effects may be obviated by the occasional exhibition of a dose of sulphate of magnesia, which, while it keeps the bowels in a soluble condition, operates as a counter-poison, by forming the inert sulphate of lead. With the same view, free use might be made of the sulphate of alumen.

From the great efficacy of white lead, prepared and applied in the manner above mentioned, a certain quantity of this article ought constantly to be kept on board our steamboats, in breweries, soap-factories, and similar establishments, where burns and scalds are of such frequent occurrence. It might thus always be employed with the least possible delay, a matter of no little moment both as respects the immediate comfort of the patient, and, in many cases also, his ultimate recovery.

Finally, although not strictly relevant, it may be mentioned here that the white lead paint promises to be of great utility in the treatment of irritable blisters, in superficial ulcerations, in excoriations of the skin, and in chilblain, or frost-bite. In the latter affection. I have, in several instances, found it to afford the most prompt relief after all the ordinary remedies had failed; it speedily allays the unpleasant itching, or tingling pain so characteristic of the disease, removes capillary congestion, and imparts new tone to the enfeebled and relaxed tissues. In one case, in which obstinate ulceration had existed for a number of years, a complete and permanent cure was effected by this remedy in less than ten days. As an application to vesicated surfaces, rendered irritable by the violent action of the fly-ointment, by the disordered state of the system, or by any other local or constitutional cause, there is no article which, in my opinion, is equal to it. The manner of applying it in these cases is the same as in burns and scalds.—*Bulletin Med. Sci., for July.*

